# Four Years of Promising Trap–Neuter–Return (TNR) in Córdoba, Spain: A Scalable Model for Urban Feline Management

**DOI:** 10.3390/ani15040482

**Published:** 2025-02-08

**Authors:** Octavio P. Luzardo, Mercedes Vara-Rascón, Agnès Dufau, Emma Infante, María del Mar Travieso-Aja

**Affiliations:** 1 Biomedical and Health Research Institute (IUIBS), University of Las Palmas de Gran Canaria, 35016 Las Palmas de Gran Canaria, Spain; marimar.travieso@ulpgc.es; 2 Faculty of Veterinary Medicine, University of Las Palmas de Gran Canaria, 35413 Las Palmas de Gran Canaria, Spain; 3 Clinical Sciences Department, University of Las Palmas de Gran Canaria, 35016 Las Palmas de Gran Canaria, Spain; 4 FdCATS, 14004 Córdoba, Spain; info@fdcats.com (M.V.-R.); agnesdufau@hotmail.com (A.D.); emmafutur@gmail.com (E.I.)

**Keywords:** trap–neuter–return, TNR, feral cats, community cats, urban ecology, urban animal populations, wildlife preservation, community engagement, animal welfare law

## Abstract

Urban free-roaming cat populations (or community cats, according to the Spanish legislation) present complex challenges, including public health risks, coexistence conflicts with residents, animal welfare, and threats to biodiversity conservation. In 2020, Córdoba, Spain, initiated one of the most extensive citywide trap–neuter–return (TNR) programs, incorporating 225 groups of cats (usually named colonies) into a meticulously structured management framework. Over four years, the program stabilized cat populations, achieving 95% sterilization coverage and effectively limiting population growth despite external challenges, including abandonment, influxes from unmanaged areas, and compensatory effects. Although the overall population showed a modest decline of 2.68%, this outcome reflects not only the recent inclusion of numerous colonies still undergoing stabilization but also the prevention of an estimated 70% population growth without intervention, as projected by our population viability analysis (PVA). Key results highlight the program’s cost-effectiveness, with an average annual cost of EUR 0.62 per person. The long-term projections from a population viability analysis (PVA) using demographic modeling suggest a potential 55% population reduction by 2028, contingent on sustained efforts. The program’s integration of citizen science, systematic data collection, and adaptive management demonstrates its scalability and replicability as a model for urban and peri-urban feline management globally. Its significance lies not only in its scope and scale but also in demonstrating the ethical and practical feasibility of large-scale TNR initiatives in urban contexts. Córdoba’s program, which predates Spain’s Animal Welfare Law 7/2023 mandating structured feline colony management plans, demonstrates a pioneering framework for compliance and innovation in urban animal management. These findings underscore the critical role of sustained interventions, community collaboration, and legal frameworks in ensuring long-term success and addressing global challenges in urban animal welfare and biodiversity conservation.

## Simple Summary

Córdoba’s trap–neuter–return (TNR) program in Spain demonstrates how citywide, well-coordinated interventions can ethically and effectively manage urban free-roaming cat populations. Over four years, the program achieved high sterilization rates across 225 colonies, stabilizing populations and addressing challenges such as abandonment and influxes from unmanaged areas. A population viability analysis (PVA) projects a significant 55% population reduction by 2028, highlighting the importance of sustained efforts, community engagement, and targeted strategies. In this study, Córdoba’s TNR initiative is positioned as a scalable and pioneering model for large-scale urban feline management in Europe.

## 1. Introduction

Urban areas worldwide are increasingly facing the challenge of managing free-roaming domestic cats, which can raise concerns for biodiversity, public health, and animal welfare. While uncontrolled feline populations have been associated with the predation of native wildlife and declines in certain bird and small mammal species across various ecosystems [1], the scale and significance of these impacts can vary. In urban environments, predation may be less frequent, as free-roaming cats often have access to human-provided food resources [2]. However, their presence can still contribute to the decline in certain species, particularly in areas with limited prey availability (e.g., parks and green spaces) [3]. In addition, their presence can still disrupt the urban wildlife behavior, altering natural activity patterns [4]. In peri-urban areas, where habitats are more interconnected, cat predation poses a greater threat to local biodiversity [5].

High densities of free-roaming cats in urban environments (up to 2500 cats/km^2^) also pose challenges such as public nuisances, zoonotic and feline disease transmission, and the contamination of public spaces [6,7,8]. These issues have led to growing global interest in humane management strategies like trap–neuter–return (TNR), which has proven effective in stabilizing cat populations when implemented with sufficient intensity and spatial coverage. However, challenges such as low sterilization rates, inadequate spatial continuity, or unmanaged immigration can hinder its success in some contexts [9,10]. Successful TNR programs require high sterilization coverage, continuous monitoring, and mitigation of external factors such as abandonment and the influx of non-neutered cats [7,8,11,12,13,14]. This approach has been increasingly adopted in many countries, including Italy, Switzerland, the United States of America, and Israel, among others, where urban TNR programs have reported reductions in cat populations and improvements in public satisfaction [7,9,15,16,17]. Social demand for ethical solutions also drives the adoption of TNR, as public opinion increasingly favors non-lethal management methods over eradication or inaction. While some animal welfare organizations criticize TNR for exposing cats to outdoor risks and advocate for their relocation to sanctuaries, other stakeholders argue against TNR on ideological and/or theoretical animal welfare grounds, favoring culling instead, presenting this option as a more humane approach [18]. Despite these polarized views, surveys consistently show strong public support for TNR as a practical and humane solution to managing free-roaming cats [19,20].

The success of urban cat management projects around the world has often been tied to collaborative frameworks involving citizens, public institutions, and non-governmental organizations. Through coordinated efforts, these stakeholders have effectively implemented TNR programs, ensured pooled resources, and aligned strategies. For example, in countries like Switzerland [17] or the United States [21,22,23], cooperation among municipal authorities, veterinary associations, and local communities has led to sustainable reductions in free-roaming cat populations. These cases underscore the importance of integrating institutional support with community engagement as a cornerstone for the long-term success of TNR programs, in other regions. Municipalities and veterinary associations play a critical role in providing organizational support and structural oversight to sustain and coordinate TNR efforts, particularly in resource-limited areas. As more cities worldwide adopt these collaborative frameworks, the need for a formal legal structure becomes increasingly evident.

Citizen science has emerged as a pivotal tool in the management of urban free-roaming cat populations, especially in programs implementing TNR strategies [24]. By empowering the public to take an active role in data collection and monitoring, citizen science expands the reach of TNR programs and ensures a continuous flow of data. In Chicago’s Humboldt Park neighborhood, the Cats in My Yard (CIMY) program demonstrated the impact of citizen involvement, achieving an 82% decrease in the local free-roaming cat population over a decade through meticulous record-keeping and coordinated TNR efforts led by a single citizen scientist [22]. Similar successes have been documented in South Australia, where the Cat Tracker project highlighted the importance of public participation in gathering valuable data on cat behavior and population dynamics, which can contribute to the development of informed management strategies [25]. Furthermore, international case studies, such as the CatScape project in Norway [26] and TNR programs in Switzerland [17], have demonstrated that citizen-led data collection provides essential insights into the spatial behaviors and ecological impact of free-roaming cats.

In Spain, the management of free-roaming cats has evolved through progressive regional and municipal efforts. The first legal references to cats as species appeared in Catalonia’s Animal Protection Law 3/1988 and Castilla-La Mancha Animal Welfare Law 7/1990 (modified in 2020) [27,28]. Free-roaming cats were specifically addressed for the first time in Asturias’s Animal Protection Law 13/2002 [29]. In 2015, Ceuta became the first region to explicitly mention TNR in its legal framework, followed by similar legislative advancements in Madrid, Galicia, and Murcia [30]. Barcelona set a national benchmark with its 2014 ordinance, which formalized TNR efforts that began in 2008 through subsidy agreements with local NGOs and the establishment of a municipal sterilization center. Notably, Spain hosted two national parliamentary forums and a regional forum in Andalusia, highlighting its commitment to ethical feline management and culminating in the enactment of the Animal Welfare Law 7/2023 [30,31,32]. This law redefines free-roaming or feral cats as “community cats,” and we will therefore employ this term consistently throughout the manuscript.

Building on this legacy, Córdoba developed one of Spain’s most structured and collaborative TNR programs. The high density of community cats in Córdoba, along with increasing public demands for ethical solutions, motivated the development of this structured and collaborative program. With the involvement of key stakeholders such as Sanitation of Cordoba, a public enterprise (SADECO), the Official College of Veterinarians, and the Federation of Animal Protection Associations of Córdoba (FAPAC), this initiative illustrates the potential of combining scientific knowledge, political commitment, and veterinary expertise to create a replicable model for managing community cat populations effectively. Córdoba’s approach highlights how localized efforts can serve as a practical demonstration of ethical feline management principles.

The objective of this study is to document the development and implementation of Córdoba’s TNR model, focusing on the collaborative framework established between citizens, institutions, and veterinarians. It evaluates the progress achieved over four years of continuous application, providing a detailed analysis of the program’s structure, strategies, and outcomes. This work aims to contribute to the global discourse on ethical and sustainable community cat management, offering a model that can be adapted to similar challenges in other regions.

## 2. Materials and Methods

### 2.1. Context and Study Area

Córdoba, a historic city in Andalusia, southern Spain, holds the distinction of having the most UNESCO World Heritage recognitions worldwide. It is home to four designated sites, as follows: the Mosque-Cathedral (1984), the Historic Center (1994), the Festival of the Patios (2012), and Medina Azahara (2018) [33]. These landmarks, along with numerous other historical and cultural treasures, establish Córdoba as a prominent global destination. This rich heritage is further complemented by sites like the Molino de la Albolafia and the Alcázar de los Reyes Cristianos both located within the Historic Center’s boundaries. Notably, these monuments and their surroundings often serve as habitats for community cat colonies—a term widely used in feline management and adopted by Spanish Animal Welfare Law 7/2023 to refer to aggregations of free-roaming cats managed under TNR programs. This highlights the complex relationship between cultural heritage and urban wildlife management.

Spanning approximately 1253 square kilometers and with a population of around 325,000 people [34], Córdoba features a mix of urban, suburban, and green spaces. Its Mediterranean climate—characterized by hot summers and mild winters—supports year-round activity for free-roaming cats. Based on our census, the vast majority of cats within the city have been accounted for; however, the number of free-roaming cats in peripheral areas remains uncertain and could increase the current census by up to 70%, as a rough estimate. Prior to the implementation of the trap–neuter–return (TNR) program, the city faced significant challenges in managing community cat populations. Colonies (we will employ this widely recognized term for aggregations of cats throughout the manuscript) often exceeded 150 individuals, resulting in issues such as poisonings, road accidents, and feline disease outbreaks. These colonies rely primarily on food provided by colony caregivers, although some also feed opportunistically on garbage or small prey. Independent sterilization efforts by citizens and animal welfare associations proved insufficient, while conflicts between cat supporters and residents who perceived them as nuisances were frequent.

The absence of an official management strategy and legal framework until late 2023 further complicated the situation. The TNR program was launched shortly after the COVID-19 lockdown, a period that exacerbated population growth due to a temporary halt in the already limited sterilization efforts.

### 2.2. Methodology: Coordination, Logistics, and Community Engagement

The trap–neuter–return (TNR) program in Córdoba was implemented within a comprehensive and collaborative coordination framework that brought together key stakeholders. The core team—comprising the project coordinators from FAPAC, representatives from the Official College of Veterinarians, SADECO, and the city council—oversaw all aspects of planning and implementation.

This multidisciplinary commission was convened at least twice a year to evaluate the program’s progress, address challenges, and refine strategies. These meetings served as a critical platform for collective decision-making and strengthened the program’s adaptive capacity.

The logistical backbone of the program was built on a highly organized approach, prioritizing sterilization efforts in geographically contiguous colonies to streamline resources and maximize impact, as recommended [14]. Mass trapping sessions were conducted in the targeted colonies, which proved crucial for rapidly stabilizing the population. The traps used were primarily Tomahawk traps (Tomahawk Live Trap Co., Ltd., Hazelhurst, WI, USA); however, complementary methods such as drop traps or large soft net traps were employed in challenging cases or high-density colonies. At the same time, the program maintained the flexibility to adapt quickly, seizing unexpected opportunities for trapping and sterilizing cats. Fieldwork presented challenges such as unexpected feline behaviors, including reluctance to enter traps despite baiting and territorial disputes among cats during trapping sessions. Additionally, coordinating large teams of volunteers in dispersed colonies required continuous adjustments and clear communication protocols.

A unique concentric approach was used to prioritize colonies for inclusion in the TNR program. The program initially focused on Córdoba’s historic urban center, an area with high tourist activity, densely populated colonies, and significant public visibility. This prioritization aimed to address locations with the highest concentration of cats and the greatest potential for human–cat conflicts. For most colonies, sterilization efforts were completed within the first year of their inclusion in the program, in line with the strategy of fully intervening in one area before advancing to the next. In subsequent years, sterilizations were primarily maintenance interventions, targeting new arrivals through abandonment or immigration rather than unsterilized individuals present during initial interventions. Gradually, the program expanded outward, reaching peri-urban areas where colonies were more geographically dispersed and included semi-owned unneutered cats. These are free-roaming cats that receive some level of care, such as feeding, from humans but do not have a defined owner and lack proper veterinary oversight, including sterilization. By 2024, most of the newly incorporated colonies were located in peripheral zones, including industrial and agricultural areas, where loose aggregations of roaming cats often exceeded 150 individuals. These large colonies posed unique challenges due to their size and limited accessibility, requiring adaptive strategies to achieve high sterilization rates effectively. Colonies were initially identified through citizen reports, caregiver registrations, and preliminary surveys conducted by local animal welfare organizations. This phased approach optimized resource allocation, enabling high-impact interventions in areas with the most urgent need while progressively expanding coverage to more remote regions.

All funding for the program, including sterilizations, logistical supplies, and veterinary costs, was provided by the City Council of Córdoba and managed by SADECO, the municipal sanitation company. FdCATS (https://www.fdcats.com/) and FAPAC provided key organizational support, but they lacked the financial resources to independently fund a program of this scale. Their roles were focused on coordination and logistical facilitation rather than direct financial contributions. It is important to note that no financial compensation was provided to volunteers, trappers, colony caregivers, or coordinators for their work. These roles were carried out on an entirely altruistic basis. Volunteers also continued to cover the cost of cat food in most cases, although they no longer had to bear the veterinary expenses for sterilizations or medical care, as they had previously covered the costsbefore the program’s implementation. Notably, neither associations nor volunteers were involved in financial transactions at any stage. All logistical needs—such as the replacement of materials, hopper feeders, shelters, and, in specific cases, the adjustment of resource locations within the cats’ home range or the provision of high-quality cat food—were communicated to the coordinating committee. Upon approval, the public sanitation company, SADECO, managed all purchases, payments, and supplies. This centralized financial management system ensured efficient and transparent resource allocation, reinforcing the program’s integrity and accountability.

A robust communication network was established primarily between the lead caregiver of each colony and the two FAPAC representatives responsible for coordination, one of whom is the first co-author of this manuscript. These representatives acted as liaisons with SADECO and the Veterinary College representatives, ensuring efficient communication across all stakeholders. Multiple WhatsApp groups facilitated constant updates and immediate responses to field challenges, enabling the team to effectively manage the complexities of working with 225 colonies.

Volunteer caregivers played a pivotal role in the program, with their participation structured and regulated to uphold high standards of colony management. Additionally, volunteer trappers were instrumental in the program’s success, conducting coordinated trapping efforts to ensure high sterilization rates and stabilize cat populations efficiently. To recruit these trappers, outreach efforts were conducted through animal welfare associations, neighborhood groups, and public events, with many volunteers also being caregivers themselves. Word-of-mouth recommendations further strengthened this network. To join the program, caregivers were required to complete an initial colony management course, after which they received an accreditation card issued by the city council. This training covered essential topics such as feeding protocols, high-intensity targeted TNR, data reporting, and best practices for managing colonies in urban and peri-urban environments. The certification not only provided caregivers with essential knowledge but also empowered them, offering formal recognition and fostering trust with local authorities. This was particularly important given the history of local caregivers facing penalties under outdated municipal ordinances. Caregivers were also required to repeat training as needed, particularly in cases where colonies showed signs of mismanagement, such as improper feeding practices, failure to report updates, or neglecting cat care. This system ensured accountability and consistency across the network of caregivers.

Community engagement served as a cornerstone for the program’s expansion. Informational meetings were organized as needed to onboard new colonies, often in collaboration with neighborhood associations, public events, and local fairs. These outreach efforts not only increased participation but also served as a platform to educate residents on responsible cat care, the prevention of abandonment, and the benefits of TNR programs, fostering trust and a sense of shared responsibility among the city’s residents. The emphasis on transparency and inclusion strengthened the program’s social foundation, ensuring broad community support for its objectives.

Adoption initiatives formed an integral part of the TNR program, managed entirely by SADECO. Eligible individuals were limited to kittens in their socialization period (under 7–9 weeks), as community cats usually cannot tolerate confinement [12,35], and, in rare cases, abandoned domestic adult cats with high sociability. These cases were reported by colony caregivers, who promptly identified and notified SADECO. Upon confirmation, the cats were removed from the streets and transferred to municipal facilities for adoption. This process not only contributed to population management but also ensured the welfare of highly vulnerable or sociable individuals.

### 2.3. Veterinary Procedures and Data Validation

Veterinary clinics participating in the TNR program followed standardized procedures to ensure consistency and quality in the sterilization process. Clinics were recruited through a framework agreement signed with the Official College of Veterinarians of Córdoba. Following this agreement, a general call was issued to all clinics, inviting them to voluntarily join the program. Each participating clinic signed an individual agreement with the College, accepting the program’s terms and conditions, including the agreed fees for each procedure. Cats were captured and transported to clinics on a rotational schedule, with two clinics assigned weekly from a pool of 23 participating clinics. A third clinic was always predesignated as backup to accommodate high capture volumes, ensuring prompt sterilization without delays. On average, clinics handled between 15 and 20 cats per week, depending on their size and the success of trapping efforts. Capture activities were conducted year round, with scheduled breaks during Holy Week, July, August, and December to account for seasonal conditions.

Surgical procedures adhered to high-quality, high-volume spay-neuter (HQHVSN) standards [36]. Each cat underwent a preoperative assessment, sterilization surgery, deworming, vaccination, ear tipping, and microchipping. The cats typically remained under observation at the veterinary clinics for an average of 24 h post-surgery. However, the final decision for discharge was made by the attending veterinarian, who ensured that the cats were fully awake, alert, and eating independently before being released. This timeframe varied depending on the complexity of the surgery and the individual condition of each cat. Cats were monitored for postoperative complications, and photographs of surgical incisions and reproductive organs (in cases of pregnancy) were recorded for documentation purposes. All cats received internal and external deworming and rabies vaccination. The program did not include trivalent vaccination due to concerns about its effectiveness without follow-up boosters, increased costs, and the potential risk of post-surgical complications, given the high volume of surgeries conducted simultaneously.

Sterilization costs were covered by SADECO, the municipal sanitation company, which reimbursed clinics based on monthly invoices. Veterinary clinics managed internal resources and staff schedules to accommodate the paperwork required for the program.

Data validation was a critical component of the program, ensuring transparency and reliability across all interventions. Veterinary clinics submitted detailed census forms alongside photographic evidence for each procedure. These records were meticulously cross-checked by FAPAC to verify their accuracy against clinic-issued invoices. Additionally, veterinarians conducted a secondary validation of the collected data, and the program coordinators carried out ongoing, exhaustive reviews of all records to ensure consistency and accuracy. Once validated, SADECO processed the corresponding payments.

Ongoing trapping after the initial mass efforts was carried out by caregivers and volunteers. Whenever new cats were identified in colonies, caregivers, often with the support of experienced trappers, promptly captured them and scheduled their sterilizations at one of the participating clinics. This strategy ensured the long-term maintenance and stability of the colonies, even in the follow-up periods spanning four years.

### 2.4. Data Management and Statistical Analyses

**Figure 1:**
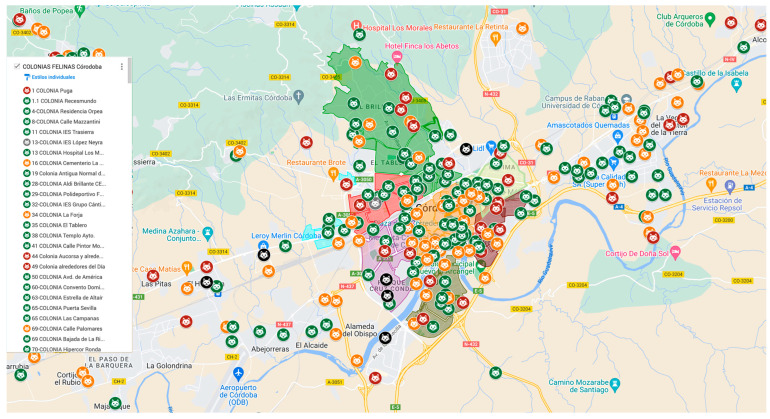
Geolocated map of community cat colonies in Córdoba, created using Google Maps. The map indicates colony sizes based on color coding: green (1–12 cats), orange (13–24 cats), red (25–60 cats), and black (more than 60 cats).

The program employed accessible technological tools and robust statistical methods to manage and analyze data effectively. Google Maps was used to geolocate cat colonies, creating interactive maps for volunteers and coordinators (Figure 1). These maps facilitated the efficient planning of trapping, sterilization, and return activities, ensuring a streamlined workflow. Data from census forms (https://www.fdcats.com/herramientas/#ficha, accessed on 10 December 2024), photographs, and videos were systematically organized into Excel spreadsheets. These census forms, provided by FdCATS, were central to standardizing data collection across colonies. The FdCATS website also hosts a range of additional materials, including detailed guidelines and resources on colony management and the TNR methodology, which were critical to the program’s structured implementation. Each colony had a dedicated folder containing individual records for each cat, including identification details, health status, and intervention history. This structured approach ensured that all information remained easily accessible, accurate, and up-to-date. These counts served as the basis for monitoring population trends over time. Caregivers and volunteers regularly documented key population dynamics, such as births, abandonments, deaths, and new arrivals, using standardized forms provided by FdCATS. These reports were submitted to FAPAC for validation, where they were cross-referenced with veterinary records to ensure accuracy and consistency in the data. In contrast, veterinary records documented each sterilization procedure, including detailed information such as the cat’s sex, health status, and photographic evidence. These records were systematically validated against clinic-issued invoices.

All statistical analyses were performed using GraphPad Prism v10.0 (Dotmatics, Boston, MA, USA). Data related to the TNR program, including population sizes, sterilization rates, and colony dynamics, were managed using Microsoft Excel v16.91. The distribution of the variables was assessed through the Kolmogorov–Smirnov test to determine normality. As most variables, including population changes and sterilization rates, did not follow a normal distribution, results are reported as medians and ranges alongside means.

Levene’s test was conducted to assess the equality of variance among groups. As it was non-significant in all cases (*p* > 0.05), the assumption of homogeneity of variance was satisfied. Statistical analyses focused on non-parametric tests due to the skewed nature of the data. Kruskal–Wallis tests were employed for comparisons among multiple groups, followed by Mann–Whitney U-tests for pairwise comparisons of total populations. The paired Wilcoxon signed-rank test was used to analyze changes in population size within colonies across different follow-up periods. This method provided robust insights into the median population changes

Specifically, the primary variables analyzed included the census data of each colony recorded annually in June. These datasets represented the number of cats per colony over different timeframes (e.g., June 2020 to June 2024 for the earliest colonies included in the program and June 2021 to June 2024 for the second group). In the PVA analysis (Section 2.5), comparisons focused on the total population recorded at the program’s inception and the projected population in theoretical scenarios.

A *p*-value of less than 0.05 (two-tailed) was considered statistically significant.

### 2.5. Population Viability Analysis (PVA) Methodology for Córdoba

We employed the Vortex 10.6.0 software [37]. This individual-based stochastic simulation model integrated local demographic and environmental parameters to predict population dynamics under various management scenarios. The initial population structure was derived from census data collected in mid-2021 (June) and subsequent years, accounting for full-year intervals. Populations were stratified into sterilized and unsterilized individuals across urban and peri-urban colonies. This approach classified cats into eight subgroups based on the year they were included in the program. Key demographic inputs, such as age distribution, reproductive rates, and mortality rates, were informed by empirical data from Córdoba’s TNR program and supplemented with findings from international studies on community cat populations [38,39,40,41].

Adoption rates, while modest, were included as a variable in the model, reflecting cases where cats were successfully rehomed. Adoption data were directly obtained from program records. Euthanasia, although minimal and applied only in cases of severe illness or injury, was incorporated as a factor influencing mortality rates, and this was modeled under the ’Harvest’ function of the software to capture its impact on population dynamics. Similarly, immigration and abandonment were incorporated as ’Population Supplements’ to reflect ongoing inflows of individuals from unmanaged areas.

Additionally, the model accounted for the specific climatic conditions in Córdoba, where elevated temperatures and year-round sunlight prevent interruptions in the reproductive cycles of female cats. Unlike traditional patterns in Northern Spain (and Europe), where reproduction typically occurs from spring to autumn, female cats in Córdoba often give birth continuously throughout the year. This leads to a higher number of litters per year, with cases observed of females nursing two litters of different ages simultaneously, as they can become pregnant within a week of giving birth.

Carrying capacity was fixed at 10,000 cats, representing an upper estimate of Córdoba’s urban ecosystem’s capacity to support free-roaming cats. This value was based on habitat availability, food resources, and space constraints, ensuring that growth projections were not artificially constrained. Although the actual population is unlikely to reach this limit due to territorial behaviors and resource competition, the estimate ensures the robustness of the model under varying conditions.

The metapopulation in this study refers to the combined population of all urban and peri-urban colonies included in Córdoba’s TNR, including the sum of both sterilized and unsterilized cats, whose dynamics were tracked through data collected by colony caregivers and validated by SADECO.

To improve the model’s accuracy, stochastic events—such as disease outbreaks, abandonment rates (estimated at 300–400 cats annually, a figure derived from national abandonment statistics [42] and adjusted proportionally based on the city’s human population size), and variability in survival and reproduction—were included. Disease outbreaks were modeled as catastrophic events with a 5% annual incidence rate and a mortality rate exceeding 50%. Sterilization efforts were also modeled as catastrophic events, with a 100% reduction in reproduction for sterilized individuals. All these data are summarized in Supplementary Table S1.

The model simulated the following two primary management scenarios: (a) Baseline (no management): this scenario assumed no intervention from 2021 to 2024, projecting population growth based on natural reproductive rates without sterilization or other controls, and (b) Future TNR (2025–2028): this scenario projected outcomes under sustained management, emphasizing increasing sterilization rates and annual maintenance efforts targeting newly identified individuals in the colonies. Each scenario was simulated over a four-year period with 1000 probabilistic iterations, ensuring robust projections. Model outputs included population size, growth rate, and extinction probabilities.

## 3. Results

### 3.1. Overview of Population Changes and 2021–2024 TNR Intervention Metrics

Since its implementation in the summer of 2020, Córdoba’s TNR program has progressively expanded to include 225 cat colonies, with follow-up periods referring to standardized annual census updates conducted as of 30 June each year. These updates include the most recent data on population changes, sterilizations, adoptions, and new colony incorporations, ensuring consistent analysis across time periods. New colonies were included annually, driven by increased trust from caregivers, who, over time, observed the program’s benefits and shared their experiences. In the most recent year (summer 2024), up to 62 new colonies were added, undergoing intensive interventions but lacking sufficient follow-up to reflect long-term outcomes. The initial total population across all colonies was 4211 cats, which decreased to 4096 by the end of the study period, representing an overall reduction of 2.7% (Table 1).

As shown in Table 1, among the 60 colonies monitored over four years, the initial population of 1763 cats showed a 2.5% decrease (95% CI: −3.5%, −1.5%). Colonies with three years of follow-up exhibited a more relevant reduction of 8.1% (95% CI: −11.1%, −4.1%), starting from an initial population of 1114 cats. In contrast, colonies monitored for only 2 years demonstrated a 5.5% increase in population size (95% CI: 2.1%, 8.8%).

When analyzed at the colony level, reductions and increases showed significant variability. For colonies with four years of follow-up, the median percentage change was −25.0% (range: −200% to +193%, 95% CI of the median: −34.8%, −11.5%), whereas for those with three years, the median was −11.1% (range: −78.9% to +177.8%, 95% CI: −20.6%, −3.8%). Colonies monitored for only two years had a median of 0% (range: −57.1% to +225%, 95% CI: 0%, 0%). These variations underline the influence of follow-up duration and colony-specific dynamics on population outcomes.

A total of 5039 sterilizations were performed across all colonies, exceeding the final population recorded in the census. This discrepancy likely reflects initial underestimations in census data, which were progressively refined as methodologies improved. It may also be attributed to factors such as abandonment, births occurring on the streets, and influxes from unmanaged areas. Of the total sterilizations, 2659 were males and 2344 were females (Table 1).

**Figure 2:**
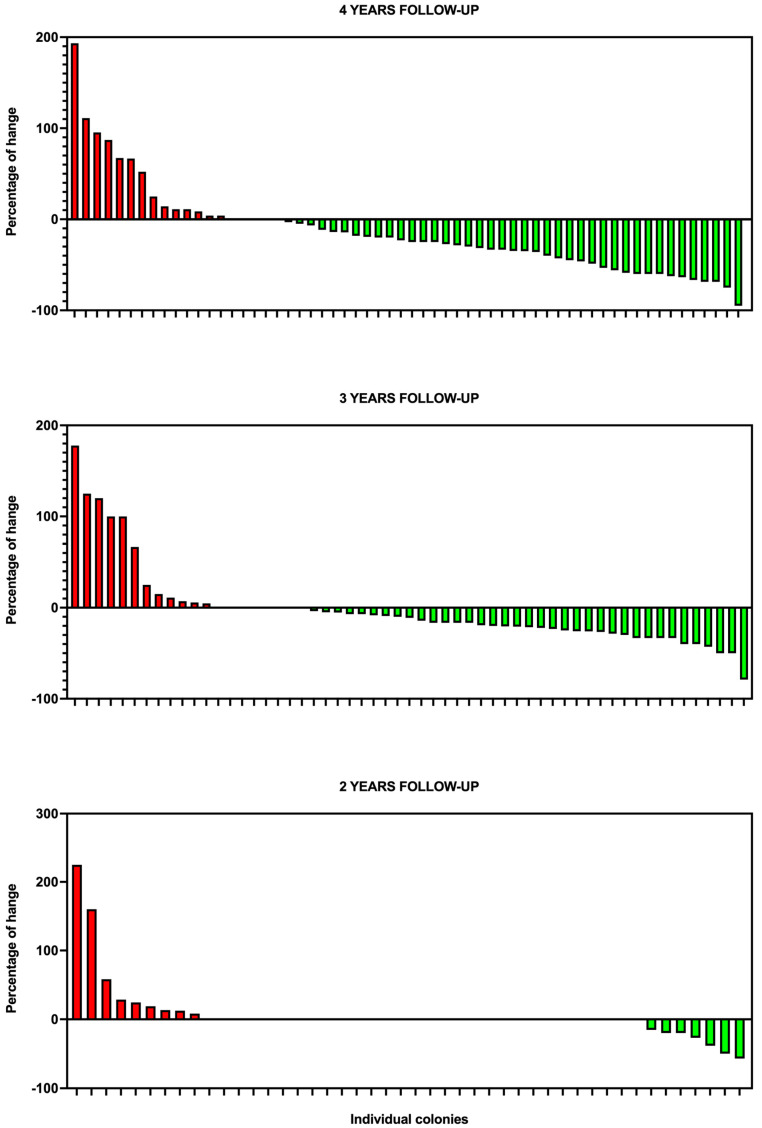
Net change in the population size of cats per colony, expressed as percentages, after 4 years (**upper panel**, colonies included in 2020), 3 years (**middle panel**, colonies included in 2021), and 2 years (**lower panel**, colonies included in 2022) of follow-up. Each bar represents an individual colony, with values calculated as the percentage difference between the population size at the start and end of the respective follow-up period. Positive values indicate an increase in population size, while negative values represent a decrease. Colonies without bars (neither red (increase) nor green (decrease)) experienced no net change in population size during the follow-up period.

The data presented in Figure 2 illustrate the changes in colony population sizes across all 225 colonies at the start and the end of the intervention period (mid-2020 to mid-2024). Each bar in the figure represents the positive or negative change in the number of individuals of an individual colony (expressed as percentage of the initial census), offering insights into the distribution of population sizes rather than aggregated totals. This approach highlights the variability and dynamics of colony populations before and after the implementation of the TNR program.

**Figure 3:**
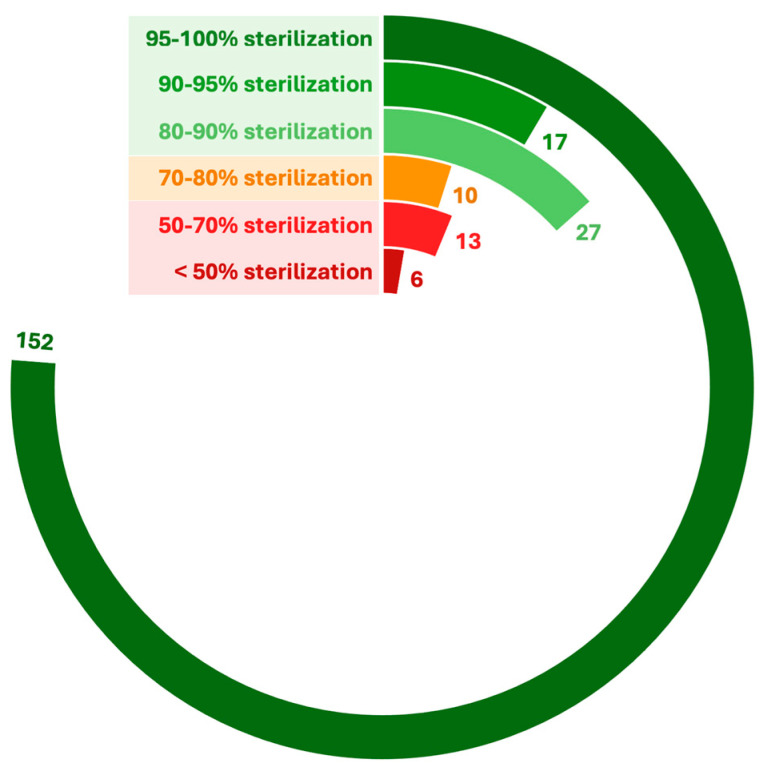
Radial chart illustrating sterilization percentages achieved across 225 colonies under Córdoba’s TNR program (2021–2024). Colonies are grouped into six sterilization rate ranges: 95–100%, 90–95%, 80–90%, 70–80%, 50–70%, and <50%. Achieving an 80% sterilization rate is widely recognized as the threshold for stabilizing a colony.

Sterilization coverage across the 225 actively managed colonies is summarized in Figure 3. The vast majority (95%) achieved sterilization levels exceeding 80%, with 152 colonies attaining rates between 95 and 100%. This high coverage reflects the program’s systematic and targeted approach to effective colony management. However, a smaller subset of colonies fell within the 50–70% sterilization range (13 colonies) or below 50% (6 colonies). Notably, some of these underperforming colonies were larger in size and/or managed by less committed caregivers highlighting areas for improvement.

### 3.2. Patterns of Population Change and Compensatory Dynamics Across Monitoring Periods

**Figure 4:**
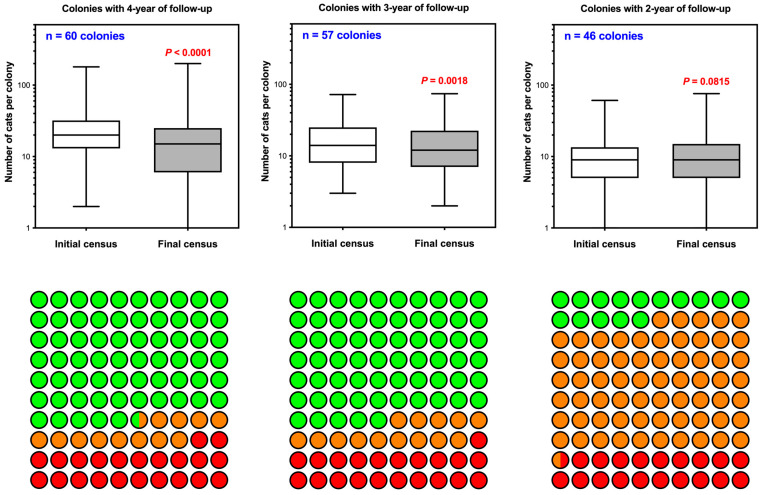
Colony dynamics across different monitoring periods in Córdoba’s TNR Program (2021–2024). The upper panels display box-and-whisker plots representing the population size changes in colonies with 4, 3, and 2 years of follow-up. Each box represents the interquartile range (IQR), with whiskers extending to the smallest and largest values within 1.5 times the IQR. The lower panels illustrate the distribution of colonies with decreasing (green), stabilized (orange), and growing (red) populations.

Monitoring data from colonies with follow-up periods of 2, 3, and 4 years illustrate distinct patterns in population changes (Table 2, and Figure 2 and Figure 4). Among the colonies with 4 years of follow-up, 70.0% showed population decreases, stabilization was observed in 8.3% of colonies, and 23.3% experienced population growth. Similarly, in colonies with 3 years of follow-up, 64.9% recorded decreases in the number of cats, 14.0% achieved stabilization, and 21.1% experienced population growth. In contrast, among colonies with only 2 years of follow-up, 15.2% showed population decreases, stabilization in the number of individuals was achieved in 65.2%, and 19.5% experienced population growth. Colonies recruited during 2024 were excluded from this trend analysis due to the limited follow-up period available for most of them.

Notably, the percentage increase in colonies experiencing growth exceeded the median decrease observed in colonies with declining populations. This highlights the variability in outcomes and underscores the challenges of achieving stabilization across all colonies.

A statistically significant reduction in the number of individuals was observed in colonies with 4 and 3 years of follow-up and consistent sterilizations of newly detected individuals during monitoring (Figure 4). In colonies with 2 years of follow-up, a decreasing trend was observed; however, the differences were not statistically significant. This outcome is likely due to the shorter time frame, which may have been insufficient for interventions to achieve stabilization.

**Figure 5:**
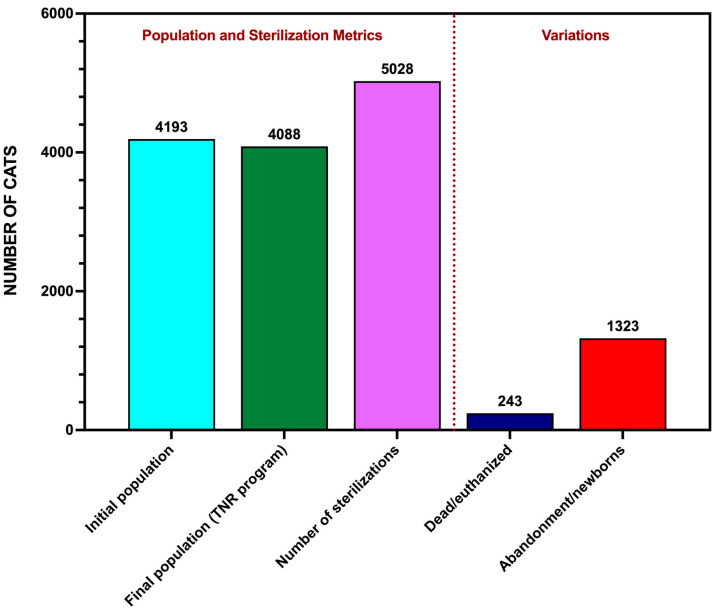
Population dynamics and sterilization metrics under Córdoba’s TNR program (2021–2024). The bar graph displays the initial and final population sizes, total sterilizations performed, recorded deaths/adoptions, and abandoned cats/new births. These data highlight the compensatory effects influencing net population changes.

The compensatory effects observed in the colonies impeded the rapid achievement of population stabilization goals (Figure 5). In colonies with less than 3 years of intervention and maintenance, the efforts primarily result in population containment rather than significant reductions. The results indicate that the number of abandoned cats and new births exceeds the total number of recorded deaths, adoptions, and population containment achieved through sterilizations.

**Figure 6:**
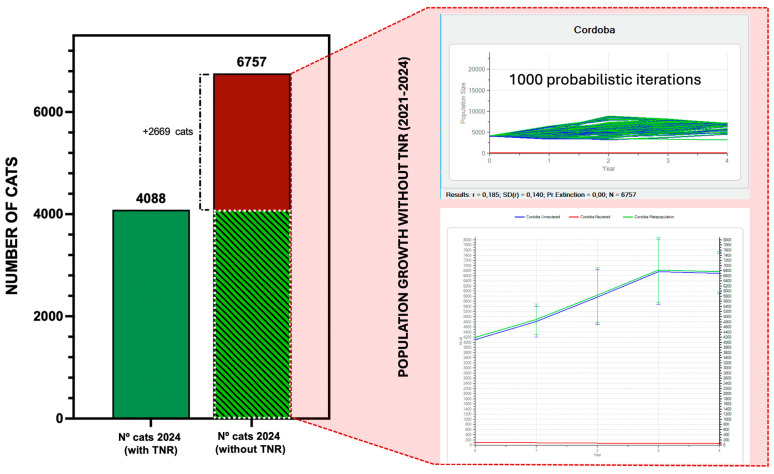
Population growth in Córdoba under hypothetical no-TNR conditions (2021–2024). The bar graph compares the observed population size in 2024 under TNR implementation (green bar) with the projected size without TNR interventions (red bar). Insets provide detailed results from population viability analysis (PVA), including growth rate and variability estimates under the unmanaged scenario.

The hypothetical scenario without TNR implementation between 2021 and 2024 highlights a stark contrast in population dynamics. Stochastic simulations (Figure 6) project that the community cats’ population in Córdoba would have increased by approximately 2669 individuals, reaching 6757 cats by 2024—a 65.3% rise from the initial 4088 cats. The projected growth rate (r = 0.185; SD(r) = 0.140; probability of extinction = 0.00) indicates a steady expansion in the absence of reproductive control. These findings underscore the critical role of TNR interventions in mitigating population growth and addressing associated challenges for animal welfare and urban biodiversity.

The economic analysis of Córdoba’s TNR program demonstrates its cost-effectiveness in controlling the community cats’ population. Over the four-year period (2021–2024), the program is estimated to have prevented the birth of nearly 3000 cats, with an average investment of EUR 0.62 per person per year, totaling EUR 2.48 per person during the study period (Table 3). This reflects an exceptionally low cost given the scale of intervention and the results achieved.

In the first year, program costs were higher due to initial investments required to establish the necessary infrastructure. These expenses included the production of shelters, hopper feeders, as well as the acquisition of equipment such as trap cages and other materials for colony management. Additionally, veterinary fees accounted for a substantial proportion of the initial costs. In subsequent years, annual expenditures varied based on material replacement, maintenance, and adjustments in program activities. Ongoing costs included veterinary services, colony feeding in certain areas, and the replacement of defective materials or equipment [12,35].

### 3.3. Long-Term Projections of Population Viability (2025–2028)

To evaluate the long-term impact of Córdoba’s TNR program, a population viability analysis (PVA) was conducted to project feline population dynamics from January 2025 to December 2028. By the end of this four-year period, colonies first included in 2021 would have been monitored for eight years, those added in 2022 for seven years, those from 2023 for six years, and those from 2024 for five years.

**Figure 7:**
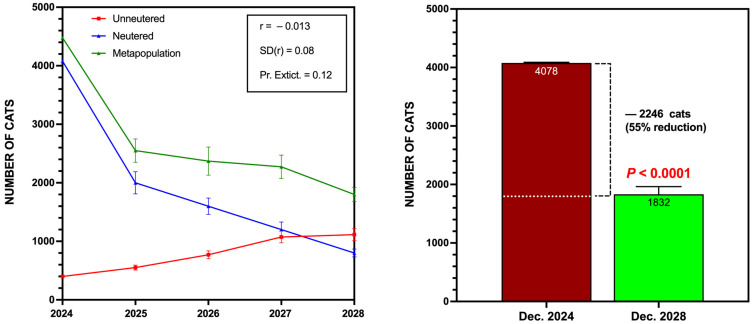
Long-term projections for Córdoba’s TNR program (2025–2028). The **left panel** shows the projected population dynamics for unsterilized, sterilized, and total metapopulations over the four-year period. The **right panel** compares the total cat population in December 2024 with the projected population by December 2028. A reduction of up to 55% of population is expected.

The results, summarized in Figure 7, indicate a significant reduction in the overall cat population, with estimates showing a decrease of up to 55% by the end of 2028. Across 1000 iterations, the average annual population decline ranged between 12% and 17%, with a wide standard deviation reflecting variability in potential outcomes under the assumed parameters [42].

It is important to note that the projections exclude new colonies that may be included into the program in the coming years, as their inclusion would require a separate analysis. These new colonies are expected to be primarily located in peri-urban and rural areas surrounding Córdoba, where the abundance of semi-owned cats, their geographic dispersion, and reduced accessibility may present additional management challenges. However, this analysis focuses specifically on evaluating the program’s effectiveness for existing colonies within the current framework.

## 4. Discussion

### 4.1. Collaborative Approaches and Transparency in Program Implementation

The implementation of the TNR program in Córdoba faced considerable obstacles from its inception in 2017. Initially, it encountered resistance from the local government, which opted for a pilot project managed by another animal welfare organization. However, limited experience in colony management posed risks to the project’s success. Recognizing these challenges, members of FAPAC contributed their expertise to ensure the project met its objectives. This early success laid the foundation for broader collaborations, involving opposition political parties, cultural associations, and the Citizens’ Movement Council. These partnerships added legitimacy and pressured the local government to adopt a comprehensive TNR strategy.

The involvement of SADECO [43], FAPAC, and the Official College of Veterinarians of Córdoba was instrumental to the program’s success. FAPAC provided the organizational backbone by managing the data collection, overseeing the methodology, and coordinating the network of volunteers, while SADECO’s logistical and financial support enhanced the program’s credibility. The College contributed technical oversight to maintain high surgical standards. This collaboration extended to 23 veterinary clinics, which rotated weekly responsibilities, ensuring flexibility and capacity to handle surplus captures when necessary. Furthermore, the TNR program strictly adhered to international guidelines and protocols, ensuring the highest standards of animal welfare and procedural rigor [12,36]. A 2023 article by FdCATS highlighted these principles, underscoring the importance of rigorous methodology, continuous volunteer training, and structured collaboration between institutions and caregivers as key factors contributing to the program’s success [44].

The approval of Spanish Animal Welfare Law 7/2023 has further reinforced the approach implemented in Córdoba. By aligning with the principles of TNR, the legislation officially recognizes this method as the preferred and ethical strategy for managing free-roaming cat populations. This alignment provides significant institutional support to the program, which had already been operational for several years prior to the law’s enactment. Despite this institutional support, the level of enforcement of Law 7/2023 has been highly uneven across Spain. While municipalities like Córdoba have fully embraced the law’s principles, others have adopted a more cautious, wait-and-see approach. Alarmingly, some regions have actively resisted the law. In the Canary Islands, for instance, various animal welfare organizations and even the national government have had to take legal action against the regional government due to noncompliance, including the promulgation of resolutions contrary to the law and the illegal confinement and euthanasia of community cats. This stark contrast highlights the challenges of ensuring uniform application of national legislation, even when it provides a clear framework for ethical and effective management practices.

Transparency and systematic data management were fundamental to the program’s long-term success. Accessible technological tools, such as Google Maps for geolocating colonies and Excel spreadsheets for organizing information, supported data collection. Standardized forms and photographic documentation of cats and surgical procedures ensured stakeholders had access to up-to-date, reliable records. These practices aligned with international guidelines [45,46], further reinforcing the program’s commitment to transparency and methodological rigor.

Volunteer engagement emerged as another cornerstone of the program’s success. Initially, many caregivers were reluctant to share colony locations or data due to distrust in municipal authorities. However, as the program consistently demonstrated its commitment to animal welfare, trust grew, fostering broader cooperation. Volunteers played a key role by implementing controlled feeding protocols to allow the trapping and to obtain reliable census data, ensuring accurate baselines for interventions.

A notable milestone in the program’s history was Córdoba’s recognition in 2022 by the Spanish government as a “City Friendly to Animals.” This distinction acknowledged the city’s leadership in ethical animal management and the transformative impact of the TNR initiative [47]. This recognition not only validated the efforts of the program’s stakeholders but also positioned it as a model for other municipalities across Spain.

The significance of community engagement in the success of free-roaming cat management techniques is well documented. A recent systematic review [20] demonstrated that high levels of community involvement, particularly in roles such as education and adoption, significantly enhance the effectiveness of TNR programs. This highlights the necessity of integrating educational campaigns and fostering public participation in TNR initiatives to ensure long-term success.

The initial challenges and subsequent achievements underscore the importance of strategic collaboration and structured organization in large-scale TNR programs. By integrating strict adherence to international protocols, systematic data management, and a resilient operational framework, the program establishes a benchmark for ethical and effective urban animal population control. These experiences provide valuable insights for other communities facing similar challenges, emphasizing the necessity of cohesive, multidisciplinary approaches.

### 4.2. Population Dynamics, Challenges, and Cost Analysis in Córdoba’s TNR Program

#### 4.2.1. Population Dynamics

The implementation of Córdoba’s TNR program over four years demonstrates the capacity of large-scale collaborative initiatives to stabilize urban feline populations. Managing 225 colonies and performing over 5000 sterilizations, the program achieved an overall reduction of 2.7% in the cat population. While the magnitude of the reduction might appear modest, it is significant given the program’s scale and the logistical challenges involved in its implementation. Notably, sterilization coverage exceeded 80% in 95% of colonies, underscoring the initiative’s operational efficiency.

Comparisons with other studies reveal both similarities and distinctions in outcomes and methodologies. In Rome, for instance, reductions in colony sizes were observed; however, the scale of the intervention—focusing on fewer colonies and a smaller urban area—differs significantly from Córdoba’s citywide approach [15]. Similarly, the targeted efforts in San Francisco achieved stabilization, yet the program operated in more confined urban settings and managed fewer colonies directly [48]. In contrast, Córdoba’s broader scale and integration of diverse habitats —including urban, peri-urban, and natural environments—introduced complexities not encountered in smaller, localized programs.

While theoretical studies provide insights into the potential impacts of management strategies [38,39], Córdoba’s program offers a real-world validation of these principles. Both emphasize the importance of high sterilization intensity and consistent follow-up to achieve long-term stabilization and population reduction. Córdoba’s program aligns closely with these findings, demonstrating how theoretical models can be effectively implemented at scale to achieve practical success.

A key challenge shared with other programs is the compensatory effect, where population growth in certain colonies offsets reductions achieved in others. Similar patterns have been reported in studies from New Zealand and Switzerland [17,49], emphasizing the influence of external factors such as abandonment and the influx of unsterilized cats on colony stability [14,17]. In Córdoba, newly included colonies, particularly those with less than three years of follow-up, exhibited population increases, highlighting the need for sustained interventions and consistent monitoring to achieve stabilization.

High sterilization rates are widely recognized as a critical factor for successful population management, as demonstrated in multiple studies [14,38]. Córdoba’s program aligns with this principle, achieving significant coverage through strategic planning and active citizen participation. The integration of citizen science for colony monitoring and the use of technological tools, such as geolocation mapping, enhanced the program’s capacity to adapt to the city’s complexity. These approaches are consistent with findings from studies in Chicago and Australia, where community engagement and data-driven methods improved intervention outcomes [22,25].

To the best of our knowledge, this is the first study to implement a citywide TNR program encompassing an entire large urban area. Previous studies have generally focused on smaller-scale interventions, targeting specific neighborhoods, university campuses, or a limited number of colonies. Córdoba’s program, therefore, establishes a precedent for the feasibility and effectiveness of large-scale TNR initiatives, providing critical insights for replication in other metropolitan contexts.

The variability in colony dynamics—with some exhibiting stabilization or reduction, while others showed growth—highlights the importance of addressing site-specific challenges. Factors such as caregiver involvement, colony size, and proximity to unmanaged areas play significant roles, as observed in both Córdoba and other studies. For example, larger colonies with less committed caregivers in Córdoba tended to have lower sterilization rates, a trend also reported in studies from Rome, San Francisco, and Massachusetts [15,23,48].

#### 4.2.2. Compensatory Effects: Challenges and Limitations

Compensatory effects, such as abandonment, new births, and influxes from unmanaged areas, present significant challenges to the implementation of TNR programs, as observed in Córdoba and other urban contexts. Despite achieving high sterilization coverage in 95% of colonies, compensatory dynamics were particularly evident in newly included colonies, where population increases offset reductions achieved in more established groups. This underscores the need for sustained follow-up and broader systemic interventions.

Similar patterns have been reported in programs such as those in Rome and Key Largo. In Rome, high abandonment rates were partially mitigated through robust community engagement and education campaigns that emphasized responsible pet ownership [15]. Meanwhile, Key Largo’s ORCAT program highlighted the importance of continuous monitoring over extended periods and successfully reduced populations despite initial setbacks caused by compensatory factors [50]. These findings align with Boone et al.’s theoretical models [38,39], which predict that external pressures—such as the abandonment and the influx of unsterilized cats—require sustained efforts to counterbalance population growth. This parallel with Córdoba’s experience emphasizes how attempts to stabilize colony populations are often hindered by these external pressures.

A unique challenge in Córdoba stems from the coexistence of urban and peri-urban colonies. The proximity to unmanaged areas allows for the continuous influx of unsterilized cats, complicating stabilization efforts. The TNR program adopted a concentric approach, starting in central, densely populated areas with high tourist activity and significant cat concentrations, and gradually expanding outward to peripheral zones. Most colonies included in 2024 were located in peripheral areas, some with as many as 150–200 individuals. These larger groups are not traditional colonies but rather loose aggregations of community cats (including semi-owned unneutered cats) across extensive areas, such as industrial zones interspersed with agricultural estates. Similar patterns have been also reported in Switzerland, where the spatial contiguity between groups of cats required intensified sterilization efforts to mitigate such dynamics [17]. Addressing these challenges demands targeted strategies, such as systematic sterilization in adjacent unmanaged areas and partnerships with neighboring municipalities to expand TNR efforts beyond city limits. However, this second phase faces unique difficulties, including a lack of caretakers, greater territorial extension, lower population density, and reduced community involvement.

Abandonment remains a persistent issue, with approximately 300–400 cats abandoned annually in Córdoba [42]. This ongoing problem intensifies compensatory dynamics, highlighting the need for measures that go beyond TNR programs. Key complementary actions include public awareness campaigns to educate citizens, stricter legal frameworks to penalize abandonment, and robust adoption initiatives. Similar strategies have proven effective in locations such as Key Largo and New Zealand, where community involvement and adoption efforts significantly mitigated compensatory effects [49,50]. Additionally, the new Spanish Animal Welfare Law 7/2023 reinforces these efforts by mandating the sterilization and registration of all owned cats over six months old in a unified national database, with exceptions only for registered breeders [32].

Córdoba’s experience highlights that compensatory dynamics represent an inherent limitation of TNR programs, particularly in large-scale implementations. Mitigating these effects requires comprehensive approaches that integrate TNR with broader societal efforts to reduce abandonment and encourage practices such as the sterilization and microchipping of pet cats, along with providing them secure environments to prevent abandonment or escape.

#### 4.2.3. Cost-Effectiveness: Evaluating the Economic Viability of TNR Programs

The economic analysis of Córdoba’s TNR program underscores its feasibility as a cost-effective strategy for managing urban community cat populations. Over the four-year period, the program operated at an average cost of EUR 0.62 per person per year, totaling EUR 2.48 per person for the entire study period. These figures reflect remarkably low expenditures compared to other TNR initiatives of similar complexity.

Programs such as those in San Francisco and Auckland demonstrate comparable economic efficiencies, though often operating on smaller scales. For instance, the San Francisco Bay Area initiative achieved stabilization within specific urban zones but incurred higher costs due to more intensive interventions per group of cats [23]. Similarly, Auckland’s pilot study reported higher per-cat costs, largely attributed to logistical challenges targeting specific urban colonies and reliance on external veterinary services [49]. In contrast, Córdoba’s integration of citizen science and partnerships with local veterinary clinics substantially reduced operational expenses.

A key strength of Córdoba’s program is its ability to balance initial infrastructure investments with sustained cost management. First-year expenses included the procurement of materials such as trap cages, feeders, and hopper feeders, as well as the establishment of veterinary partnerships. These upfront costs were amortized over subsequent years, enabling a steady decline in annual expenses without compromising the program’s effectiveness.

Adoption initiatives further contributed to cost-effectiveness by reducing population growth while imposing minimal financial burden. This approach aligns with practices observed in Key Largo and Switzerland, where adoption programs played a central role in population management and public engagement [17,50].

The long-term projections further highlight the economic benefits of sustained TNR interventions. Without the program, Córdoba’s community cat population was estimated to increase by 65.3% over four years, exacerbating challenges for animal welfare and urban biodiversity. By preventing the birth of nearly 3000 cats, the program not only stabilized populations but also reduced potential costs associated with unmanaged growth. These outcomes align with theoretical models that emphasize the cost-effectiveness of early, sustained interventions in preventing exponential population growth and its associated economic and ecological consequences [38,39]. By bridging theoretical projections with real-world applications, Córdoba’s TNR program illustrates the tangible benefits of integrating scientific models into practical implementation, setting a benchmark for urban population management strategies.

### 4.3. Long-Term Projections and Implications: Analyzing the PVA Results for 2025–2028

The population viability analysis (PVA) conducted for Córdoba’s TNR program provides critical insights into the potential long-term outcomes of sustained sterilization and population management efforts. Projections indicate a significant reduction of up to 55% in the overall feline population by 2028, assuming continued interventions and adherence to the program’s operational framework. This projected decline aligns with the annual reduction rates observed during the first four years of the program, reinforcing the efficacy of long-term commitment to TNR strategies. The stochastic model’s standard deviation of 19.8% indicates a wide range of potential outcomes, providing flexibility for variability. Nevertheless, even under the most conservative estimates, the model predicts a substantial reduction of at least 35% in population size, further highlighting the program’s overall success.

The data emphasize the compounded benefits of consistent sterilization efforts combined with effective long-term monitoring. Colonies included in the program since 2021 are expected to show the most pronounced reductions, illustrating the cumulative impact of sustained efforts. Similar trends have been documented in other long-term studies, such as the ORCAT program in Key Largo, which achieved population stabilization and reductions of up to 86% over two decades of continuous intervention [50]. These findings underscore the importance of patience, persistence, and ongoing management in achieving meaningful results in TNR programs—particularly in urban contexts with high population densities and complex dynamics.

To ensure the accuracy of the PVA predictions and maximize the program’s impact, strategies such as adoption initiatives, public awareness campaigns, and efforts to mitigate abandonment rates remain critical. The recent enactment of new animal welfare legislation presents a pivotal opportunity to strengthen these strategies. This legislation mandates sustained sterilization efforts—including for owned cats—and introduces comprehensive measures to address abandonment [32,51]. By institutionalizing these efforts, the legislation not only secures the long-term sustainability of Córdoba’s TNR program but also establishes a more cohesive and resilient framework for addressing the challenges of urban feline population management. Studies from New Zealand and Switzerland [17,25], emphasize that combining legal frameworks with public engagement strategies provides a structured roadmap for strengthening TNR initiatives. Following these models, Córdoba’s program demonstrates how integrated legal and public engagement strategies can enhance population management and promote sustainable outcomes [12,18].

### 4.4. Integrating Conservation and Management: The Iconic Example of the Albolafia Mill Colony

While the focus in this study is predominantly on the urban management of community cats, the principles and methodologies applied are equally relevant to colonies located in natural or historically significant sites [51]. A notable example of this integration is the colony at the Molino de la Albolafia, an iconic Arab mill by the Guadalquivir River, prominently featured on the coat of arms of Córdoba. Situated near the renowned Mosque-Cathedral of Córdoba—one of the most visited monuments in Spain—this colony exemplifies the importance of incorporating all types of habitats—urban, peri-urban, and natural—into a cohesive and humane management strategy that ensures both ecological integrity and animal welfare.

Through the implementation of the TNR program, the cat population in this colony has decreased from 18 to 6 individuals, demonstrating the program’s capacity to stabilize and gradually reduce colony sizes. In addition to sterilization, a key measure contributing to this success was the adoption of ad libitum feeding with high-protein kibble, as recommended by some authors [52]. Notably, this feeding protocol was implemented only after completing the initial sterilization of 100% of the cats in the colony (n = 18), thus avoiding the challenges that ad libitum feeding could pose during trapping efforts. This continuous access to high-quality food, combined with sterilization, appeared to reduce the cats’ predatory behavior. Prior to the intervention, bird remains were frequently observed along the pedestrian pathway, and cats often scavenged food from tourists, leading to litter accumulation around the site. Following the program’s implementation, these issues have been notably mitigated, resulting in cleaner surroundings and reduced dependence on hunting.

**Figure 8:**
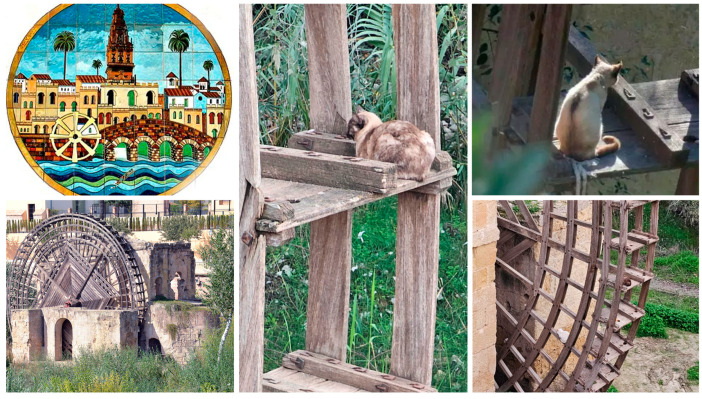
Community cats at the Molino de la Albolafia, Córdoba. The left side of the image shows a view of the mill (1137). At the top, the city of Córdoba’s coat of arms prominently features this emblematic feature. The remaining composition includes photographs of some of the six cats currently residing in the colony.

Moreover, these cats have become an attraction for tourists, fostering a positive perception of community cats and illustrating how humane interventions can coexist with heritage conservation efforts. This example underscores how Córdoba’s TNR program successfully integrates animal welfare, ecological preservation, and cultural heritage, providing a replicable model for other cities facing similar challenges (Figure 8).

### 4.5. Limitations of the Study

Despite the promising outcomes and robust design of Córdoba’s TNR program, several limitations must be acknowledged.

First, the modest overall population reduction observed during the initial four years reflects the progressive inclusion of new colonies into the program. Many of these colonies have not yet reached the stabilization phase, which can take several years of sustained intervention. This highlights the importance of long-term commitment and underscores the risk of prematurely evaluating the success of similar programs based solely on early results.

Another significant limitation is the impact of external factors such as abandonment, influxes from unmanaged areas, and compensatory effects, all of which can undermine population control efforts. Although these challenges were partially mitigated through high sterilization coverage and community engagement, their persistence highlights the inherent difficulty of managing urban and peri-urban feline populations. Without broader systemic measures—such as the strict application of the legal framework, public awareness campaigns, and enforcement of responsible pet ownership—the program’s long-term success could be jeopardized.

The reliance on citizen science and volunteer participation, while a strength in many respects, also introduces variability in data quality and program efficiency. Inconsistent caregiver commitment and logistical constraints in certain colonies have resulted in disparities in sterilization rates, underscoring the need for continued training, oversight, and support for volunteers.

Finally, the projections derived from the population viability analysis (PVA) are inherently subject to uncertainty, as they depend on assumptions about demographic parameters and environmental conditions. Any deviations from these assumptions—such as changes in abandonment rates, unforeseen disruptions in program funding, or logistical constraints—could significantly alter the program’s projected outcomes.

## 5. Conclusions

The Córdoba TNR program establishes a benchmark as one of the most comprehensive and remarkable large-scale initiatives for the ethical and effective management of urban and peri-urban feline populations. Its distinctiveness lies in its extensive geographic scope, the scale of the managed population, and the sustained, well-organized efforts implemented over four years. The program demonstrates meticulous planning, robust organization, dedicated funding, and the unwavering commitment of all stakeholders, including volunteers, veterinary clinics, animal protection NGOs, and public institutions. These elements collectively ensure high sterilization rates across 225 colonies, covering 95% of the managed population, while progressively incorporating new colonies into the framework.

Over four years, the program achieved stabilization of cat populations and mitigated growth, despite challenges such as abandonment, immigration from unmanaged areas, and compensatory effects. The modest overall population decline of 2.7% during the study period reflects the progressive inclusion of new colonies that have not yet reached stabilization. However, the program has prevented the birth of nearly 3000 new cats according to the population viability analyses (PVA), which moreover project a significant 55% reduction in the feline population by 2028, provided the program maintains its current operational intensity and coverage. These projections underscore the efficacy of sustained interventions and highlight Córdoba’s program as a replicable model for other urban settings.

Cost-effectiveness has been another cornerstone of the program, operating at an average annual cost of EUR 0.62 per person. This low financial burden demonstrates that large-scale TNR initiatives can achieve meaningful outcomes with efficient resource allocation and community collaboration. The integration of citizen science, systematic data collection, and adaptive management has been pivotal in enhancing the program’s scalability and replicability.

The recent enactment of Spain’s Animal Welfare Law 7/2023 provides an opportunity to institutionalize and sustain these efforts. This legislation mandates structured feline colony management plans, including comprehensive censuses, registration, and the intensive application of the TNR method. By aligning with these legal requirements, the Córdoba TNR program serves as a model for compliance, offering critical insights for other municipalities as they work to fulfill their obligations under the new legislation. This framework ensures not only the long-term sustainability of Córdoba’s program but also establishes a foundation for scaling similar efforts nationwide.

As the program continues to evolve, future evaluations will determine whether the anticipated success materializes or whether additional strategies need to be designed and refined. Ongoing assessment will be essential to ensure the program’s long-term impact and adaptability, providing a roadmap for ethical and evidence-based management of urban animal populations.

## Figures and Tables

**Table 1:** Population dynamics and sterilization outcomes across different follow-up periods in Córdoba’s TNR program (2021–2024).

Follow-Up (Years)	Number of Colonies	Initial Census	Final Census	Change	% Variation	Sterilized Males	Sterilized Females	Total Sterilized ^a^
4	60	1763	1708	−53	−2.5%	1128	1136	2300 (+31%)
3	57	1114	1024	−90	−8.1%	642	619	1261 (+13%)
2	46	549	579	+30	+5.5%	266	256	522 (−5%)
1	62	785	785	0	0.00%	618	328	946 (+20%)
**Totals/Average**	**225**	**4211**	**4096**	**−113**	**−2.7%**	**2659**	**2344**	**5039 (+20%)**

^a^ The numbers between parentheses represent the ratio of total sterilized cats to the initial census size, reflecting the intensity of sterilization efforts across follow-up periods.

**Table 2:** Colony dynamics based on monitoring periods: decreasing, stabilizing, and growing colonies (2021–2024).

	4-Year Follow Up				
	Number of Colonies (% of Total)	Average Change (%)	Range (%)	Median Change (%)	Median Number of Cats/Colony
**Colonies that decrease**	42 (70.0%)	−40.3%	−100.0% to −3.1%	−34.9%	23
**Colonies Stabilized**	5 (8.3%)	—	—	—	17
**Colonies Growing**	14 (23.3%)	53.7%	4.1% to 193.3%	52.2%	47
	**3-Year Follow-Up**				
	**Number of Colonies (% of Total)**	**Average change (%)**	**Range (%)**	**Median Change (%)**	**Median Number of Cats/Colony**
**Colonies that decrease**	37 (64.9%)	−24.1%	−78.9% to −3.8%	−21.4%	23
**Colonies stabilized**	8 (14.0%)	—	—	—	10
**Colonies growing**	12 (21.1%)	63.2%	4.7% to 177.8%	45.8%	17
	**2-Year Follow-Up**				
	**Number of Colonies (% of Total)**	**Average Change (%)**	**Range (%)**	**Median Change (%)**	**Median Number of Cats/Colony**
**Colonies that decrease**	7 (15.2%)	−32.6%	−57.1% to −15.4%	−26.9%	11
**Colonies stabilized**	30 (65.2%)	—	—	—	10
**Colonies growing**	9 (19.5%)	61.1%	8.3% to 225.0%	24.6%	18

**Table 3:** Economic summary of Córdoba’s TNR program: cost analysis per cat, colony, and person (2021–2024).

Years of Monitoring	Number of Colonies	Total Program Cost (€)	Cost per Cat (€)	Cost per Person/Year (€)
4	60	332.320	195.48	1.02
3	57	295.530	294.80	0.91
2	46	122.320	208.48	0.38
1	62	212.160	264.25	0.16
**Total/Average**	**225**	**1162.330**	**240.75**	**0.62**

## Data Availability

The data supporting the reported results are available upon request from the corresponding author. Due to ethical and privacy considerations, the data are not publicly accessible.

## Supplementary Materials

The following supporting information can be downloaded at: https://www.mdpi.com/article/10.3390/ani15040482/s1, Table S1: Key Parameters and Assumptions for PVA in Córdoba’s Community Cat Population.
