# Supplementary material for: Four Years of Promising Trap–Neuter–Return (TNR) in Córdoba, Spain: A Scalable Model for Urban Feline Management
Source: Animals (Basel). 2025 Feb 8;15(4):482. doi: 10.3390/ani15040482 (PMC11851652; doi:10.3390/ani15040482)
Supplement: Supplementary file 1 [file animals-15-00482-s001.zip › animals-3436897-supplementary.pdf]

Table S1. Key Parameters and Assumptions for PVA in Córdoba's Community Cat Population.

Parameter	Value/Assumption	Justification
**Carrying Capacity**	10,000 cats	Fixed to reflect the maximum estimated population that Córdoba's urban ecosystem could theoretically support.
**Reproductsive Rates**	3 litters/year per breeding female	Reflects continuous reproduction due to Córdoba's Mediterranean climate (no seasonal interruption).
**Litter Size**	3.5 kittens per litter (average)	Empirical data from field observations and the literature for urban cat populations.
**First-Year Mortality Rate**	50% (both sexes)	High mortality rate due to environmental stressors such as predation, disease, and starvation.
**Reproductive period (females)**	6 months to 6 years	Represents the age range during which females are reproductively active in most cases
**Reproductive period (males)**	5 months to 7.5 years	Represents the age range during which males are reproductively active in most cases
**Mortality Rate (Subsequent)**	10% (females), 15% (males) per year	Reflects higher male mortality due to territorial disputes and roaming behaviors.
**Maximum lifespan**	8 years	Based on observations from the TNR program, most cats die before reaching 6 years old, although some exceptions exist
**Sterilization Impact**	100% reduction in reproduction for sterilized individuals	Modeled as a catastrophic event, completely halting reproduction in sterilized subpopulations.
**Disease Outbreaks**	Viral incidence: 5% annually; mortality: >50%	Modeled as stochastic catastrophic events to simulate potential epidemics (e.g., calicivirus, panleukopenia).
**Immigration and Abandonment**	14% annually	Modeled as 'Population Supplement' to reflect ongoing abandonment and migration from unmanaged areas.

**Adoption rate**	1.5% annually	Included as a 'Harvest' parameter reflecting adoption as a minor but consistent population decrease factor.
**Euthanasia**	<1% annually. Based on veterinary records	Includes humane euthanasia recorded during monitoring. Modeled under 'Harvest'
